# The Effect of Lipotoxicity on Renal Dysfunction in a Nonobese Rat Model of Metabolic Syndrome: A Urinary Proteomic Approach

**DOI:** 10.1155/2019/8712979

**Published:** 2019-12-06

**Authors:** Irena Markova, Denisa Miklankova, Martina Hüttl, Petr Kacer, Jelena Skibova, Jan Kucera, Radislav Sedlacek, Tereza Kacerova, Ludmila Kazdova, Hana Malinska

**Affiliations:** ^1^Centre for Experimental Medicine, Institute for Clinical and Experimental Medicine, 14021 Prague, Czech Republic; ^2^Czech University of Life Sciences, 16500 Prague, Czech Republic; ^3^Czech Centre for Phenogenomics, Institute of Molecular Genetics of the Czech Academy of Sciences, 25250 Vestec, Czech Republic; ^4^Department of Chemistry, University College London, London WC1H 0AJ, UK

## Abstract

**Introduction:**

The development of metabolic syndrome-associated renal dysfunction is exacerbated by a number of factors including dyslipidemia, ectopic deposition of lipids and their toxic metabolites, impairment of lipid metabolism, and insulin resistance. Renal dysfunction is also affected by the production of proinflammatory and profibrotic factors secreted from adipose tissue, which can in turn directly impair kidney cells and potentiate insulin resistance. In this study, we investigated the manifestation of renal lipid accumulation and its effect on renal dysfunction in a model of metabolic syndrome—the hereditary hypertriglyceridemic rat (HHTg)—by assessing microalbuminuria and targeted urinary proteomics. Male Wistar control rats and HHTg rats were fed a standard diet and observed over the course of ageing at 3, 12, and 20 months of age.

**Results:**

Chronically elevated levels of triglycerides in HHTg rats were associated with increased levels of NEFA during OGTT and over a period of 24 hours (+80%, *P* < 0.01). HHTg animals exhibited qualitative changes in NEFA fatty acid composition, represented by an increased proportion of saturated fatty acids (*P* < 0.05) and a decreased proportion of n-3 PUFA (*P* < 0.01). Ectopic lipid deposition in the kidneys of HHTg rats—triglycerides (+30%) and cholesterol (+10%)—was associated with markedly elevated microalbuminuria as ageing increased, despite the absence of microalbuminuria at the young age of 3 months in these animals. According to targeted proteomic analysis, 3-month-old HHTg rats (in comparison to age-matched controls) exhibited increased urinary secretion of proinflammatory parameters (MCP-1, IL-6, IL-8, *P* < 0.01) and decreased urinary secretion of epidermal growth factor (EGF, *P* < 0.01) before manifestation of microalbuminuria. Elevation in the urinary secretion of inflammatory cytokines can be affected by increased relative expression of MCP-1 in the renal cortex (*P* < 0.05).

**Conclusions:**

Our results confirm dyslipidemia and ectopic lipid accumulation to be key contributors in the development of metabolic syndrome-associated renal dysfunction. Assessing urinary secretion of proinflammatory cytokines and epidermal growth factor can help in detecting early development of metabolic syndrome-associated renal dysfunction.

## 1. Introduction

Metabolic syndrome (MetS) and prediabetes are accompanied by a number of metabolic disturbances, cardiovascular and hemodynamic complications, and renal dysfunction. Subjects with metabolic syndrome are at increased risk of developing chronic kidney disease (CKD) and diminished renal function [1, 2]. A recent prospective study demonstrated that more than one-third of subjects with metabolic syndrome had markedly declined renal function measured as the estimated glomerular filtration rate (GFR) [1]. Accumulating evidence indicates that MetS and insulin resistance are independent risk factors for the development and progression of kidney disease [3].

The close relationship between MetS and increased incidence of CKD might be explained by their common pathogenetic mechanisms, such as chronic inflammation, oxidative stress, and insulin resistance. Ectopic lipid accumulation and disturbances in lipid metabolism [4] may represent other important causes of metabolic disturbance and contribute to renal lipid metabolism. Apart from factors such as inflammation, hemodynamic parameters, and adipokines, renal lipotoxicity has been proposed as playing a crucial role in the relationship between kidney disease and MetS [5].

Alterations in kidney dysfunction can be induced by dyslipidemia. Besides increased plasma triglycerides and LDL-C, important roles are played by elevated plasma NEFA and their impaired metabolism. NEFA critically contribute to the process of lipotoxicity in tissues, generate lipotoxic intermediators, promote insulin resistance, and potentiate the production of proinflammatory cytokines [6]. Although the general role of dyslipidemia remains poorly defined, higher levels of triglycerides and LDL-C and decreased levels of HDL-C appear to be associated with greater risks of albuminuria and declining GFR [7].

Renal dysfunction can also be affected by the production of proinflammatory and profibrotic factors secreted from adipose tissue, directly impairing kidney cell function and further potentiating insulin resistance. Inflammation may mediate the development of renal fibrosis and glomerulosclerosis in MetS [2]. Perirenal adipose tissue, a part of abdominal visceral fat, may have a close relationship to renal damage. In one study of obese rats [8], an increase in perirenal fat was related to microalbuminuria and inflammation activation. However, the exact role of perirenal adipose tissue on kidney disorder and dysfunction is not completely understood.

Since MetS-associated renal dysfunction can start before the onset of hypertension and diabetes, early detection seems to be important. Microalbuminuria is currently the most reliable predictor of declining renal function, but its predictive power is limited by poor sensitivity and specificity. New biomarkers like urinary proteomics are now being used to identify kidney dysfunction in its earlier state or independently of microalbuminuria [9]. In addition, urinary proteins and peptides may reflect changes in protein expression, deposition, and turnover in the kidney, while providing more information about the pathophysiology of the disease.

Most of the existing studies on renal lipotoxicity in animal models have been performed in high-fat diet or genetically induced obesity. To investigate the role of lipid disorders and perirenal adipose tissue on kidney function, we used a nonobese rat model of metabolic syndrome and prediabetes, hereditary hypertriglyceridemic rats (HHTg). Originating from Wistar rats, this strain exhibits dyslipidemia, insulin resistance, fatty liver, mild hypertension, and low-grade chronic inflammation in the absence of hyperglycemia or obesity [10]. Compared to Wistar control rats, the strain of HHTg rats during aging does not gain weight just like control rats, and in young age, the both strains of rats have comparable adiposity.

## 2. Materials and Methods

### 2.1. Animals and Diet

All experiments were performed in agreement with the Animal Protection Law of the Czech Republic (311/1997) in compliance with European Community Council recommendations (86/609/ECC) for the use of laboratory animals and approved by the Ethics Committee of the Institute for Clinical and Experimental Medicine, Prague.

Wistar control rats and HHTg rats—serving as a model of insulin resistance and metabolic syndrome—were fed a standard chow diet (23% protein, 43% starch, 7% fat, 5% fibre, and 1% vitamin and mineral mixture; Bonagro, Czech Republic) and examined at 3, 12, and 20 months of age. Rats were housed in stainless steel wire-mesh cages and held under temperature- (22°C) and humidity-controlled conditions at a 12 h/12 h light-dark cycle. At all times, the animals had free access to food and water. Rats were randomly divided into experimental groups (*n* = 8‐10). At the beginning of the study, body weight, serum glucose, and triglycerides were measured. At the end of the experiment, rats were sacrificed by decapitation (not to affect the parameters of insulin sensitivity) in a postprandial state (to better reflect metabolic changes associated with prediabetes and metabolic syndrome). The collected tissue samples were immediately frozen in liquid nitrogen. At the end of the experiment the blood was collected into tubes without the addition of anticoagulants. Aliquots of serum, urine, kidney, and adipose tissue samples were stored at -80°C for analysis.

### 2.2. Analytical Methods/Biochemical Analysis

Serum levels of triglycerides, glucose, total cholesterol, and NEFA were measured using commercially available kits (Erba Lachema, Czech Republic and Roche Diagnostics, Germany). Qualitative fatty acid composition of the NEFA lipid class was analysed by gas chromatography as previously described [11]. For determination of serum or urinary creatinine and uric acid, an enzymatic spectrophotometric method was used (available kits from Roche Diagnostics, Germany).

Serum insulin and adiponectin concentrations were determined using the Rat Insulin ELISA kit (Mercodia AB, Sweden) and the Rat Adiponectin ELISA kit (MyBioSource, USA). Serum IL-6 and MCP-1 were measured using rat ELISA kits (BioSource International, USA and eBioscience-Bender MedSystems Biocenter, Austria). For the oral glucose tolerance test (OGTT), blood glucose was determined after intragastric administration of a glucose load (300 mg/100 g b.wt.) and following overnight fasting. Blood was drawn from the tail before the glucose load at 0 min and thereafter at 30, 60, and 120 min.

To determine triglycerides and cholesterol in tissues, samples were extracted in chloroform/methanol. The resulting pellet was dissolved in isopropyl alcohol, after which triglyceride content was measured by enzymatic assay (Erba Lachema, Czech Republic).

Albumin levels in urine were analysed using a high-performance liquid chromatography method with UV-Vis detection according to Contois et al. [12] and then adjusted for creatinine concentration.

### 2.3. Metabolomic and Proteomic Biomarkers

Oxidative stress (4-HNE, MDA, 8-isoprostan) and inflammatory (IL-6, IL-8, MCP-1, heparan sulfate) proteomic biomarkers were analysed using a method consisting of a pretreatment step, solid phase extraction (SPE) for rapid and effective isolation of biomarkers, and liquid chromatography tandem mass spectrometry (LC-MS/MS) detection. For multimarker screening, we performed two types of analysis: the first to detect compounds containing amino groups and the second to detect compounds with aldehyde and carboxylic groups [13]. Urinary protein candidate biomarkers were analysed by a combination of immunomagnetic separation and MALDI-TOF mass spectrometry. Custom-designed antibody microarrays were used to confirm MS data by comparing the relative content of different analytes in the samples [14].

### 2.4. Relative mRNA Expression

Total RNA was isolated from the renal cortex and epididymal and perirenal adipose tissues using RNA Blue (Top-Bio, Czech Republic). Reverse transcription and quantitative real-time PCR analysis was performed using the TaqMan RNA-to C_T_ 1-Step Kit and TaqMan Gene Expression Assay (Applied Biosystems, USA) on the ViiA™ 7 Real-Time PCR System (Applied Biosystems, USA). Relative expressions of SCD, IL-6, and MCP-1 were determined after normalisation against *β*-actin as an internal reference and calculated using the 2^-*ΔΔ*Ct^ method. Results were run in triplicate.

### 2.5. Histological Assessment

10 *μ*m thick cryosections from the kidney were cut using the CM1950 cryostat (Leica, Germany) and mounted on Superfrost Plus™ glass slides (Thermo Fisher Scientific, USA). After drying and fixation in 4% formaldehyde, the sections were stained with Oil Red O staining according to standard protocol with hematoxylin counterstaining. The presence of neutral lipids in the kidney was evaluated and documented by a board-certified histopathologist using the Axio Scope.A1 microscope (ZEISS, Germany) at 100x to 400x magnification and the AxioCam ICc 5 camera (ZEISS, Germany).

### 2.6. Statistical Analysis

Our aim was to highlight urinary proteomic biomarkers before the onset of microalbuminuria in a model of metabolic syndrome. To this end, we used one-way and two-way ANOVA to test for the combined effect of the strain and age. For variables showing evidence of age (3 m or 20 m), we used the Bonferroni post hoc test (*P* < 0.05) for multiple comparisons to determine whether urinary proteomic biomarkers had been significantly altered in the HHTg strain compared to the WISTAR strain. Statistics for basal metabolic parameters were calculated in the same way. Student's *t*-test was used to compare variables of fatty acid composition in the NEFA lipid class and for serum parameters at the age of 3 months before the onset of microalbuminuria. All results are expressed as mean ± standard error of the mean (SEM). Statistical analysis was performed using BMDP Statistical Software.

## 3. Results

### 3.1. Basal Metabolic Parameters

HHTg rats across all age groups exhibited less weight gain in comparison to age-matched Wistar control rats. Although the strain of HHTg rats is nonobese, these animals exhibited greater relative weight of epididymal (indicated as the adiposity index) and perirenal fat pads (Table 1). Compared to age-matched controls, HHTg rats exhibited markedly increased serum triglyceride concentrations and decreased HDL-C; however, there were no differences in serum cholesterol (Table 1). All serum lipid parameters worsened as ageing increased in both rat strains.

**Figure 1:**
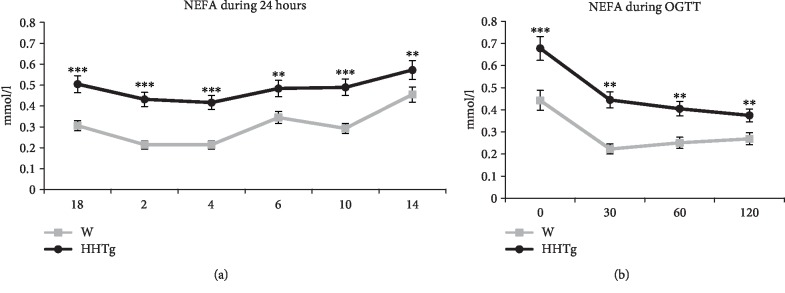
Concentrations of NEFA over 24 hours (a) and during OGTT (b) in HHTg rats (*n* = 6) compared to Wistar controls (*n* = 6) at 3 months of age. Data are expressed as mean ± SEM and analysed by one-way ANOVA; ∗∗ denotes *P* < 0.01 and ∗∗∗ denotes *P* < 0.001. HHTg: hereditary hypertriglyceridemic rats, W: Wistar control rats, NEFA: nonesterified fatty acids, OGTT: oral glucose tolerance test.

Serum levels of NEFA in HHTg rats chronically increased as ageing increased, during OGTT, and over a period of 24 hours (+80%, *P* < 0.01) (Figure 1). In the case of the NEFA lipid class, animals exhibited qualitative changes in NEFA fatty acid composition, represented by an increased proportion of saturated fatty acids (SFA) (*P* < 0.05) and a decreased proportion of n-3 PUFA (*P* < 0.01) (Table 2). Between serum adipocytokines and inflammatory markers, only leptin and MCP-1 were increased in HHTg rats at the age of 3 months compared to age-matched controls (Table 3).

### 3.2. Renal Lipids in relation to Microalbuminuria and Histological Observation

**Figure 2:**
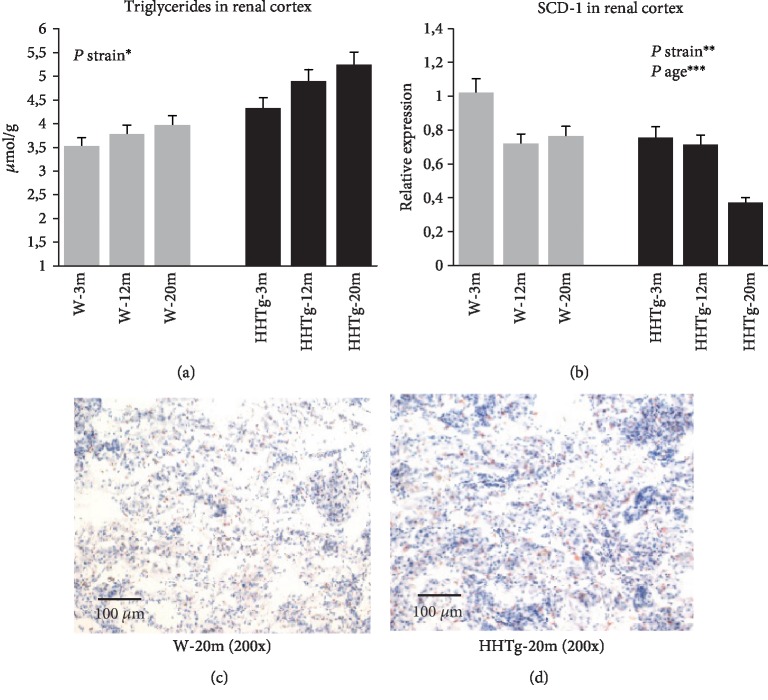
Concentrations of triglycerides (a) and relative gene expression of SCD-1 (b) in the renal cortex. Histological comparison of the kidneys of Wistar control (c) and HHTg (d) rats at 20 months of age. Data are expressed as mean ± SEM and analysed by two-way ANOVA; ∗ denotes *P* < 0.05, ∗∗ denotes *P* < 0.01, and ∗∗∗ denotes *P* < 0.001. HHTg: hereditary hypertriglyceridemic rats, W: Wistar control rats, SCD-1: stearoyl-CoA desaturase.

As shown in Figure 2, the amount of accumulated triglycerides in the kidneys of HHTg rats was significantly higher compared to age-matched controls. HHTg rats also exhibited decreased relative expression of stearoyl-CoA desaturase-1 (SCD-1), a key lipogenic enzyme. There were no significant differences in renal cholesterol concentrations between HHTg and age-matched control animals (data not shown). The presence of neutral lipids, localised predominantly in the renal tubulus and partly in the glomeruli of HHTg rats, was verified based on histological observation. Rare, albeit almost negligible, evidence of the presence of neutral lipids was observed in age-matched controls (Figure 2).

### 3.3. Microalbuminuria and Proteomic Analysis

**Figure 3:**
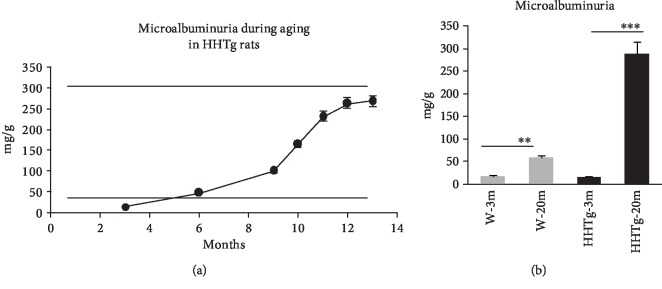
Microalbuminuria levels in relation to ageing in HHTg rats (a) (*n* = 6) and compared to Wistar control rats (b) (*n* = 8). Data are expressed as mean ± SEM and analysed by two-way ANOVA; ∗∗ denotes *P* < 0.01 and ∗∗∗ denotes *P* < 0.001. HHTg: hereditary hypertriglyceridemic rats, W: Wistar control rats.

As shown in Figure 3, levels of microalbuminuria rose markedly as HHTg rats aged, despite the absence of microalbuminuria at the young age of 3 months. In comparison to age-matched controls, targeted proteomic-based analysis revealed elevated urinary secretion of proinflammatory parameters (MCP-1, IL-6, and IL-8) in 3-month-old HHTg rats before manifestation of microalbuminuria (Table 4). Increased urinary secretion of heparan sulfate—proteoglycan of extracellular matrix—can also activate inflammatory processes. On the other hand, the secretion of epidermal growth factor (EGF)—a marker of tubular mass—significantly decreased at a young age in HHTg rats without the onset of microalbuminuria. Compared to MDA, urinary secretion of 4-HNE—a sensitive parameter of lipoperoxidation—significantly increased in HHTg rats at the young age of 3 months (Table 4).

### 3.4. Relative Expression of Proinflammatory Parameters in Perirenal and Epididymal Adipose Tissue and the Renal Cortex

**Figure 4:**
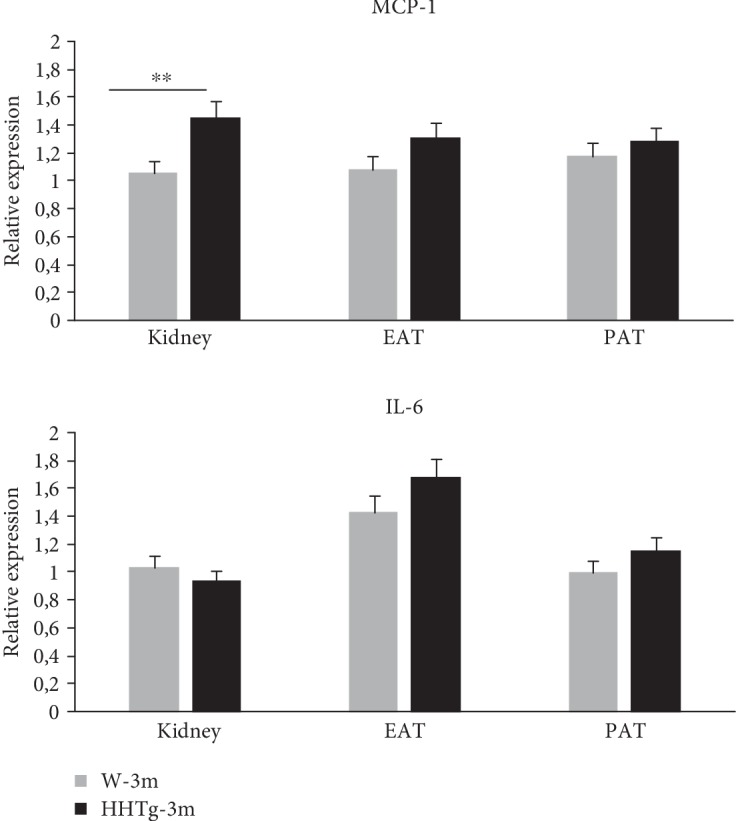
Relative gene expression of proinflammatory markers MCP-1 and IL-6 in the renal cortex, epididymal adipose tissue (EAT), and perirenal adipose tissue (PAT) in HHTg rats (*n* = 8) and Wistar control rats (*n* = 8) at 3 months of age and before microalbuminuria onset. Data are expressed as mean ± SEM and analysed by one-way ANOVA; ∗∗ denotes *P* < 0.01. HHTg: hereditary hypertriglyceridemic rats, W: Wistar control rats, MCP-1: monocyte chemoattractant protein-1, IL-6: interleukin 6.

Based on the results of targeted proteomic analysis, we investigated gene expression of MCP-1 and IL-6 in the kidneys as well as in perirenal and epididymal adipose tissue (Figure 4). In contrast to age-matched controls, relative expression of MCP-1 in the renal cortex was significantly elevated in HHTg rats at the age of 3 months and before manifestation of microalbuminuria. However, there were no differences in gene expression of IL-6 in the renal cortex at a young age in rats. In perirenal adipose tissue, there were no differences in gene expression in either cytokine despite the elevated relative weight of perirenal adipose tissue.

**Figure 5:**
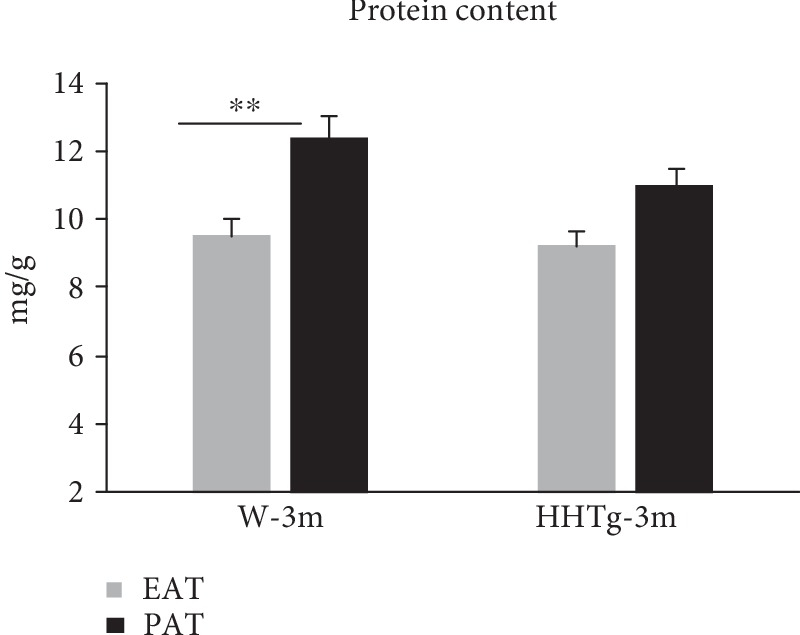
The protein content in epididymal adipose tissue (EAT) and perirenal adipose tissue (PAT) in HHTg rats (*n* = 8) and Wistar control rats (*n* = 8) at 3 months of age. Data are expressed as mean ± SEM and analysed by one-way ANOVA; ∗∗ denotes *P* < 0.01. HHTg: hereditary hypertriglyceridemic rats, W: Wistar control rats.

In the renal cortex and epididymal adipose tissue, we also observed markedly elevated gene expression of MCP-1 and IL-6 in both rat strains as ageing increased. In contrast to epididymal adipose tissue, protein content in perirenal adipose tissue significantly increased in Wistar control rats as well as in HHTg rats (Figure 5).

## 4. Discussion

Although medical understanding of the exact role of dyslipidemia in the initiation and progression of kidney disease is incomplete, the results of our study support the hypothesis that kidney functions are negatively affected by dyslipidemia, leading to qualitative and quantitative alterations in blood lipids and lipoproteins. In our model of MetS and insulin resistance—the HHTg rat—dyslipidemia was represented by chronically elevated levels of triglycerides and NEFA and decreased levels of HDL-C, all characteristic features of MetS- and T2D-associated dyslipidemia. Resulting from adipose tissue insulin resistance and increased lipolysis, elevated levels of NEFA in HHTg rats promote ectopic lipid deposition in different tissues. This can in turn directly impair cell functions and lead to the generation of toxic lipid metabolites such as diacylglyceroles and ceramides, whose interference with intracellular signalling pathways may cause and promote insulin resistance and other metabolic disorders.

NEFAs and their aggravated metabolism can affect the function of podocytes [6], which are highly susceptible particularly to saturated fatty acid lipotoxicity. In one study that used cultures of human podocytes, palmitic acid induced insulin resistance and podocyte dysfunction [15]. Aggravated renal insulin sensitivity has been also observed in the glomeruli of obese as well as diabetic rats [16]. In addition to elevated levels of NEFA, qualitative changes in the fatty acid profile of the NEFA lipid class can contribute to NEFA-induced lipotoxicity. In the present study, chronically elevated NEFA concentrations in HHTg rats were accompanied by qualitative alterations in the NEFA lipid class, characterised by an increased proportion of SFA and a decreased proportion of n-3 PUFA. Some studies have reported a higher proportion of SFAs in serum NEFA in obese and type 2 diabetic patients [17, 18]. In our study, the proportion of palmitoleic and dihomo-*γ*-linoleic fatty acids markedly increased in correlation with the development of insulin resistance. Albumin-bound NEFA are filtered through the glomeruli and reabsorbed by the proximal tubulus, a process that can directly and aggressively contribute to renal damage, promote renal insulin resistance, cause renal lipid accumulation, and activate proinflammatory pathways.

Renal triglyceride accumulation is commonly observed in animal models of diet- and genetic-induced obesity [19, 20]. In the present study, we observed triglyceride accumulation in the kidneys of HHTg rats independently of the presence of obesity. Based on histological observation, we found evidence of the presence of neutral lipids in the renal tubular cells and glomeruli of HHTg rats. Our results confirm that renal damage by lipotoxicity is a complex process not only associated with microvascular complications but also with the functions of the glomerulus and tubulus.

Alterations in renal lipid metabolism can contribute to ectopic lipid deposition in the kidney, while reduction of SCD-1 gene expression may contribute to the accumulation of SFA in the kidney and is understood to be associated with SFA-bound albumin-mediated renal lipotoxicity. In the renal cortex of HHTg rats, we observed decreased relative mRNA expression of SCD1, a key lipogenic enzyme. In another study, diabetic mice fed a high-fat diet also exhibited decreased SCD1 renal expression [21]. Downregulation of SCD1 can lead to the accumulation of saturated fatty acids, subsequently impairing kidney functions. Thus, any imbalance in the lipid metabolism of enzymes and genes involved in lipid oxidation and regulation (SREBP, PPAR*γ*) can contribute to renal lipid accumulation. Decreased expression of SCD1 may play an important pathogenic role in lipotoxicity-induced renal damage. Accordingly, recent studies have suggested that SCD1 may become a novel therapeutic target for protecting against proteinuria in diabetic nephropathy and reducing SFA-induced lipotoxicity [21].

A key mechanism involved in intrarenal lipid-induced kidney damage is endoplasmic reticulum stress, which disturbs endoplasmic reticulum homeostasis, decreases folding capacity, and leads to the accumulation of unfolded and misfolded proteins in cells [22]. It should be noted that, in their connection with ectopic lipid deposition, NEFA and triglycerides exert different effects. Indeed, lipid accumulation can have a partly protective effect, provided nonoxidised fatty acids are only deposited in the form of neutral triglycerides and lipotoxic intermediates fail to generate.

Apart from insulin resistance and renal lipotoxicity, other factors also affect kidney function in a metabolic syndrome state. Kidney function worsens with age, while hypertension can have a considerably negative effect on renal function. Our previous results showed that HHTg rats can exhibit mild hypertension (150/110 mm Hg at the age of 6 months) in positive correlation with triglyceride plasma levels [23]. Further, the accumulation of lipoperoxidation products in the intimal part of the arterial wall in HHTg rats resulted in decreased NO synthase activity and cGMP concentrations, in turn impairing endothelial vasorelaxation. Impairment of NO-dependent relaxation in the aorta of HHTg rats was not associated with increased activity of the renin-angiotensin-aldosterone system. Therefore, we postulate that hypertension in these animals has a minor effect on renal function compared to the severe hypertriglyceridemia and chronically elevated NEFA.

In the present study, dyslipidemia and renal lipid accumulation in HHTg rats were accompained by an age-dependent gradual elevation of albuminuria, with microalbuminuria appearing at the age of 6 months in these animals. Microalbuminuria is considered a traditional risk marker for the diagnosis and prognosis of declining renal function. Some studies demonstrated an association between insulin resistance and microalbuminuria, and MetS may contribute to the manifestation of albuminuria in patients with diabetes mellitus [24, 25].

However, the determination of microalbuminuria lacks of sufficient specificity and sensitivity, while the positive predictive value of microalbuminuria has been reported to be only 50% in albuminuric patients [26]. Recent studies have revealed new promising urinary markers for patients at high risk of renal dysfunction independently of, or before, the onset of microalbuminuria. Based on the urinary proteomic-based analysis in this study, we identified increased urinary secretion of a sensitive lipoperoxidation marker (4-HNE), a tubular profibrotic marker (EGF), extracellular matrix parameters, and proinflammatory parameters (MCP-1, IL6, IL8) in HHTg rats before microalbuminuria onset. Other studies have found increased urinary secretion of proinflammatory cytokines in T1 diabetic patients [27], T2 diabetic patients [28], and obese individuals [29]. In a prospective clinical study of diabetic patients with overt nephropathy [30], urinary secretion of MCP-1 and IL-6 had an independent and additive effect on proteinuria in predicting the rate of declining renal function. Our study is the first to confirm the increased secretion of proinflammatory parameters before microalbuminuria onset in relation to renal lipotoxicity using a nonobese model of metabolic syndrome.

We observed that increased urinary secretion of MCP-1 and IL-6 in HHTg rats was accompanied by decreased urinary secretion of EGF, another promising marker of early renal dysfunction. Low urinary EGF may be useful as a predictive marker of progressively declining kidney function, especially in patients with normoalbuminuria [31]. Some authors contend that a combination of EGF with MCP-1 in the form of urinary EGF/MCP-1 may actually be a better predictor of eGFR decline than using one marker alone [32, 33]. According to our results, urinary markers may be better at predicting the risk and progression of renal damage than serum inflammatory markers.

Proteomic analysis may enhance our understanding of the pathogenesis of kidney dysfunction. Based on the results of proteomic analysis, we investigated gene expression of inflammatory markers. Compared to epididymal and perirenal adipose tissue, gene expression of MCP-1 in the renal cortex increased in HHTg rats before microalbuminuria onset, which suggests that inflammatory pathways are not only directly activated in the kidney but also play an important role during the early developmental phase of lipotoxocity-induced kidney dysfunction. MCP-1 is secreted by glomerular and tubular endothelial cells, promoting macrophage accumulation in the kidney and activating renal inflammation. Thus, urinary secretion of MCP-1 reflects damage in glomerular as well as in tubular cells.

Elevated secretion of heparan sulfate in HHTg rats likely exacerbates inflammation. Increased MCP-1 bound to heparan sulfate, a proteoglycan found in the extracellular matrix, can lead to the activation of increased expression of MCP-1 and L-selectin in cells [34]. Based on our observations of HHTg rats, urinary MCP-1 seems to be causally linked to kidney damage and may be useful as an early marker for identifying lipotoxicity-induced renal damage.

While the IL-6 cytokine can activate an inflammatory response, increase fibronectin expression, enhance endothelial permeability, and stimulate proliferation of mesangial cells, it is also directly involved in the pathogenesis of insulin resistance [35]. And as IL-6 activates gluconeogenesis and the hepatic secretion of triglycerides, its urinary secretion may reflect more than renal inflammation alone. The sources of IL-6 are macrophages (40%) and also vascular cells (60%). In addition, 70% of urinary proteins originate from kidney tissue, and the remaining 30% derive from plasma that can reflect the situations in other tissues. That may be the reason why elevated urinary secretion of IL-6 was not associated with the changes in gene expression in the kidney.

Numerous studies report the distribution of visceral fat to be a major risk factor for kidney disease [1]. Perirenal fat thickness has emerged as an independent predictor of renal dysfunction in patients with diabetes [36] and in obese patients [37]. In the present study, we observed no significant changes in the gene expression of inflammatory cytokines in perirenal adipose tissue despite increased weight of perirenal adipose tissue.

Perirenal adipose tissue thus probably exerts a paracrine effect, where the release of excess NEFA increases renal NEFA intake, leading to the activation of inflammatory pathways in the kidney. It is also possible that the partially protective effects of perirenal fat can prevent their conversion to lipotoxic intermediates, especially during early phases of renal impairment development.

Altogether, then, the interplay between altered lipid metabolism, chronic inflammation, and insulin resistance seems to be important in the pathogenesis of renal microvascular complications in MetS [38]. But while the kidney actively participates in metabolic changes leading to its impairment, renal lipid accumulation would seem to be more a mediator than the result of renal injury per se.

In conclusion, our results indicate that dyslipidemia and ectopic lipid accumulation, even in the absence of obesity, play key roles in the development of metabolic syndrome-associated renal dysfunction. Increased concentrations of NEFA and higher proportions of saturated fatty acids in NEFA may be involved in this process.

Based on our assessment, urinary secretion of proinflammatory cytokines and epidermal growth factor positively correlated with metabolic changes in the kidney, a finding that may be useful in detecting the early development of metabolic syndrome-associated renal dysfunction.

## Figures and Tables

**Table 1:** Basal metabolic characteristics of Wistar (*n* = 8) and HHTg rats (*n* = 8) observed over the course of aging at 3, 12, and 20 months of age.

	Wistar			HHTg			*P*_1_ value	*P*_2_ value
	3 m	12 m	20 m	3 m	12 m	20 m		
Body weight	407 ± 5	635 ± 28	663 ± 21	304 ± 5	478 ± 14	566 ± 12	^∗∗∗^	n.s.
Adiposity index	1.090 ± 0.057	1.457 ± 0.151	1.316 ± 0.195	1.161 ± 0.044	2.531 ± 0.043	2.534 ± 0.070	^∗∗∗^	^∗∗∗^
Perirenal adipose tissue	0.971 ± 0.061	1.760 ± 0.067	1.946 ± 0.202	1.386 ± 0.308	3.110 ± 0.107	2.437 ± 0.111	^∗∗∗^	^∗^
Nonfasting glucose Insulin	7.2 ± 0.3 0.198 ± 0.047	6.6 ± 0.1 0.213 ± 0.023	6.4 ± 0.6 0.278 ± 0.045	7.8 ± 0.2 0.307 ± 0.053	8.2 ± 0.3 0.286 ± 0.046	7.6 ± 0.7 0.353 ± 0.081	^∗∗^ ^∗∗^	^∗^ n.s.
AUC_0-120_	660 ± 43	705 ± 16	679 ± 26	737 ± 26	1036 ± 35	1011 ± 55	^∗∗^	^∗^
Serum triglycerides	1.55 ± 0.14	1.84 ± 0.18	2.07 ± 0.51	5.45 ± 0.45	5.33 ± 0.72	9.82 ± 0.79	^∗∗∗^	^∗∗∗^
Serum cholesterol	1.74 ± 0.11	2.33 ± 0.16	4.12 ± 0.72	1.52 ± 0.05	1.69 ± 0.09	2.71 ± 0.26	^∗∗^	n.s.
HDL-C	1.35 ± 0.13	1.57 ± 0.11	2.26 ± 0.27	0.56 ± 0.05	0.66 ± 0.03	0.45 ± 0.06	^∗∗∗^	^∗∗∗^
NEFA Hepatic triglycerides	0.209 ± 0.021 7.25 ± 0.45	0.360 ± 0.025 9.99 ± 1.04	0.334 ± 0.027 5.08 ± 0.40	0.401 ± 0.037 11.03 ± 0.77	0.661 ± 0.053 20.73 ± 1.23	0.473 ± 0.072 15.96 ± 1.57	^∗∗∗^ ^∗∗∗^	^∗^ ^∗∗^

Values are given as mean ± SEM, *n* = 8 for each group; ∗ denotes *P* < 0.05, ∗∗denotes *P* < 0.01, and ∗∗∗ denotes *P* < 0.001. Body weight (g), adiposity index (mg/g), perirenal adipose tissue (mg/g), glucose, triglycerides, cholesterol, AUC_0-120_ (mmol/l), HDL-C and NEFA (mmol/l), hepatic triglycerides (*μ*mol/g), insulin (nmol/l). *P*_1_ value: probability reflecting the effect of the strain. The analysis was performed using BMDP statistical software by two-way ANOVA and by the Bonferroni post hoc test for multiple comparisons. *P*_2_ value: probability reflecting the combined effect of the strain and ageing. The analysis was performed using BMDP statistical software by two-way ANOVA and by the Bonferroni post hoc test for multiple comparisons.

**Table 2:** Fatty acid composition for the NEFA lipid class in Wistar (*n* = 8) and HHTg rats (*n* = 8) at 3 months of age.

	W-3 m (% of total fatty acids)	HHTg-3 m (% of total fatty acids)	*P* value
16:00	10.751 ± 0.908	11.318 ± 1.129	n.s.
18:00	13.234 ± 0.725	18.024 ± 1.872	<0.05
16:1n7	0.516 ± 0.089	1.299 ± 0.202	<0.01
18:2n6	14.632 ± 0.940	12.861 ± 1.044	n.s.
20:3n6	0.883 ± 0.100	3.040 ± 0.435	<0.001
20:4n6	13.157 ± 1.007	13.726 ± 0.868	n.s.
20:5n3	3.540 ± 0.305	1.889 ± 0.530	<0.05
22:5n3	7.193 ± 0242	3.609 ± 0.437	<0.001
SFA	24.222 ± 0.515	29.554 ± 2.602	<0.05
MUFA	12.151 ± 0.835	13.540 ± 1.678	n.s.
n-6 PUFA	40.166 ± 0.608	38.041 ± 1.695	n.s.
n-3 PUFA	23.216 ± 0.664	18.445 ± 1.239	<0.01

Values are given as mean ± SEM; **n** = 8 for each group. The relative concentration of each fatty acid was calculated as a proportion of all of the fatty acids detected: percentage of total fatty acids. Statistical significance was calculated by Student's **t**-test. W: Wistar control rats, HHTg: hereditary hypertriglyceridemic rats, SFA: saturated fatty acids, MUFA: monounsaturated fatty acids, PUFA: polyunsaturated fatty acids.

**Table 3:** Serum adipokines, proinflammatory parameters, and basal renal markers in Wistar control (*n* = 8) and HHTg rats (*n* = 8) at the age of 3 months before microalbuminuria onset.

	W-3 m	HHTg-3 m	*P* value
Adiponectin (*μ*g/ml)	6.05 ± 0.34	5.87 ± 0.41	n.s.
Leptin (*μ*g/ml)	2.65 ± 0.21	3.60 ± 0.17	<0.05
TNF-*α* (pg/ml)	2.62 ± 0.31	3.12 ± 0.49	n.s.
IL-6 (pmol/l)	13.8 ± 1.9	14.5 ± 2.8	n.s.
MCP-1 (pmol/l)	172 ± 23	245 ± 35	<0.05
Creatinine (mmol/l)	16.7 ± 3.4	17.3 ± 1.6	n.s.
Uric acid (*μ*mol/l)	71.6 ± 4.9	84.7 ± 10.6	n.s.

Values are given as mean ± SEM; *n* = 8 for each group. Statistical significance was calculated by Student**'**s **t**-test. W**:** Wistar control rats, HHTg**:** hereditary hypertriglyceridemic rats, TNF-*α ***:** tumour necrosis factor *α*, IL-6**:** interleukin 6, MCP-1**:** monocyte chemoattractant protein-1.

**Table 4:** Urinary proteomic biomarkers in Wistar (*n* = 8) and HHTg rats (*n* = 8) observed at 3 months of age before microalbuminuria onset and after microalbuminuria manifestation at 20 months of age.

	3 m		*P*_3 m_ value	20 m		*P*_20 m_ value	*P*_W/HHTg_ value
	W	HHTg		W	HHTg		
Malondialdehyde (ng/ml)	23.3 ± 0.7	25.4 ± 1.5	n.s.	25.0 ± 0.9	27.8 ± 1.2	n.s.	^∗^
8-Isoprostan (pg/ml)	22.8 ± 1.6	26.2 ± 1.6	n.s.	22.6 ± 1.6	25.3 ± 1.6	n.s.	n.s.
4-Hydroxynonenal (ng/ml)	15.7 ± 1.0	19.8 ± 0.6	^∗^	16.0 ± 1.9	21.2 ± 0.6	n.s.	^∗∗^
IL-6 (pg/ml)	48.7 ± 1.4	82.3 ± 4.2	^∗∗∗^	53.7 ± 0.7	81.3 ± 4.8	^∗∗∗^	^∗∗∗^
IL-8 (pg/ml)	20.9 ± 1.7	44.9 ± 1.6	^∗∗∗^	25.9 ± 1.6	44.6 ± 1.8	^∗∗∗^	^∗∗∗^
MCP-1 (ng/ml)	1.7 ± 0.1	3.4 ± 0.2	^∗∗∗^	2.0 ± 0.1	3.3 ± 0.2	^∗∗∗^	^∗∗∗^
EGF (ng/ml)	4.7 ± 0.3	2.0 ± 0.2	^∗∗∗^	4.8 ± 0.2	2.0 ± 0.1	^∗∗∗^	^∗∗∗^
*α*1-Antitrypsin (ng/ml)	12.1 ± 0.7	22.0 ± 1.3	^∗∗∗^	14.1 ± 1.0	21.7 ± 1.5	^∗∗∗^	^∗∗∗^
Heparan sulfate (*μ*g/ml)	0.07 ± 0.01	0.28 ± 0.01	^∗^	0.13 ± 0.01	0.30 ± 0.01	^∗^	^∗∗∗^

Values are given as mean ± SEM; *n* = 8 for each group; ∗ denotes *P* < 0.05, ∗∗ denotes *P* < 0.01, and ∗∗∗ denotes *P* < 0.001. *P*_3 m_: probability reflecting the effect of the strain at 3 months of age and were analysed by two-way ANOVA and by the Bonferroni post hoc test. *P*_20 m_: probability reflecting the effect of the strain at 20 months of age and were analysed by two-way ANOVA and by the Bonferroni post hoc test. *P*_W/HHTg_: probability reflecting the effect of the strain and were analysed by two-way ANOVA and by the Bonferroni post hoc test. IL-6: interleukin 6, IL-8: interleukin 8, MCP-1: monocyte chemoattractant protein-1, EGF: epidermal growth factor.

## Data Availability

The data used to support the findings of this study are included within the article.

## Abbreviations

AGEs: Advanced glycation end products
CKD: Chronic kidney disease
eGFR: Estimated glomerular filtration rate
EGF: Epidermal growth factor
4-HNE: 4-Hydroxynonenal
MetS: Metabolic syndrome
NEFA: Nonesterified fatty acids
GSH: Reduced form of glutathione
GSSG: Oxidised form of glutathione
IL-6: Interleukin 6
T2D: Type 2 diabetes
MDA: Malondialdehyde
MCP-1: Monocyte chemoattractant protein-1
PPAR: Peroxisome proliferator-activated receptors
SCD-1: Stearoyl-CoA desaturase-1
SFA: Saturated fatty acid
SREBP: Sterol regulatory element-binding protein
TNF-*α*: Tumour necrosis factor *α*
TGF-*β*: Transforming growth factor *β*
NRF2: Nuclear factor-erythroid 2-related factor-2.
